# An *in vivo* genetic screen in *Drosophila* identifies the orthologue of human cancer/testis gene *SPO11* among a network of targets to inhibit *lethal(3)malignant brain tumour* growth

**DOI:** 10.1098/rsob.170156

**Published:** 2017-08-30

**Authors:** Fabrizio Rossi, Cristina Molnar, Kazuya Hashiyama, Jan P. Heinen, Judit Pampalona, Salud Llamazares, José Reina, Tomomi Hashiyama, Madhulika Rai, Giulia Pollarolo, Ismael Fernández-Hernández, Cayetano Gonzalez

**Affiliations:** 1 Cell Division Group, Institute for Research in Biomedicine (IRB Barcelona), The Barcelona Institute of Science and Technology, Baldiri Reixac, 10, 08028 Barcelona, Spain; 2 Institució Catalana de Recerca i Estudis Avançats (ICREA), Passeig Lluís Companys, 08010 Barcelona, Spain

**Keywords:** l(3)mbt, *Drosophila*, malignant growth, cancer testis antigens

## Abstract

Using transgenic RNAi technology, we have screened over 4000 genes to identify targets to inhibit malignant growth caused by the loss of function of *lethal(3)malignant brain tumour* in *Drosophila in vivo*. We have identified 131 targets, which belong to a wide range of gene ontologies. Most of these target genes are not significantly overexpressed in mbt tumours hence showing that, rather counterintuitively, tumour-linked overexpression is not a good predictor of functional requirement. Moreover, we have found that most of the genes upregulated in mbt tumours remain overexpressed in tumour-suppressed double-mutant conditions, hence revealing that most of the tumour transcriptome signature is not necessarily correlated with malignant growth. One of the identified target genes is *meiotic W68* (*mei-W68*), the *Drosophila* orthologue of the human cancer/testis gene *Sporulation-specific protein 11* (*SPO11*), the enzyme that catalyses the formation of meiotic double-strand breaks. We show that *Drosophila mei-W68/SPO11* drives oncogenesis by causing DNA damage in a somatic tissue, hence providing the first instance in which a *SPO11* orthologue is unequivocally shown to have a pro-tumoural role. Altogether, the results from this screen point to the possibility of investigating the function of human cancer relevant genes in a tractable experimental model organism like *Drosophila.*

## 1. Background

Anatomical and physiological differences between humans and relatively simple model organisms like *Drosophila melanogaster* are many and major, and may preclude the modelling of some key aspects of malignant growth. However, basic cellular processes are conserved among cells that, regardless of the species, derail from their normal course of development, grow out of control, become immortal, invasive and kill the host.

Among the wide range of tumour models that can be experimentally induced in *Drosophila*, some are frankly malignant neoplasms [1–4]. These experimental tumour models offer an unprecedented opportunity to carry out high-content *in vivo* screens at a speed and with a level of coverage that are not yet attainable in vertebrate model systems. A number of genetic and chemical screens carried out in the last few years substantiate such a potential [5–11].

Tumours caused by loss of function of *lethal(3)malignant brain tumour* (*l(3)mbt*) (henceforth referred to as mbt tumours) originate during development in the larval neuroepithelium, spread over the optic lobes, and may eventually invade the ventral nerve cord [12,13]. Upon allografting, mbt tumours grow, invade the abdomen and kill the host in a few weeks. Indeed, mbt tumours can undergo limitless rounds of allografting and are, therefore, immortal [12].

Gene expression profiling studies have revealed that mbt tumours upregulate hundreds of genes and have helped to define an mbt tumour signature (MBTS) that uniquely identifies these tumours apart from other larval brain tumour types [12]. A significant fraction of the MBTS comprises genes whose expression and function in wild-type individuals is mostly constrained to the germline. Three of these genes have been shown to be essential for mbt tumour growth [12]. Unscheduled expression of germline genes in somatic tumours is not unique to *Drosophila*; it has been abundantly reported in human oncology studies where such genes are collectively referred to as cancer/testis (CT) or cancer-germline (CG) genes [14,15]. Remarkably, some of the germline genes upregulated in mbt tumours are orthologues of catalogued human CT genes. Moreover, meta-analysis of expression profile datasets shows that upregulation of the human orthologues of mbt tumour-associated germline genes is common in human cancers [16].

In this study, we have made use of the *l(3)mbt* model and the extant collection of transgenic RNAi *Drosophila* lines to interrogate the genome for functions that are essential for tumour growth. The screen was designed to identify targets that upon depletion severely curtail mbt tumour growth while still allowing for larval development. We have screened a random selection of 4000 genes, representing about a third of the total number of protein coding sequences in *Drosophila*, together with a selected group of about 200 candidate genes. Altogether, our results identify a network of potential targets to inhibit malignant growth. Among them are genes that had not been linked to cancer before, which may provide leads for the development of new strategies to repress malignant growth, as well as others like *Translationally Controlled Tumour Protein* (*Tctp*), *tudor* (*tud*) and *mei-W68* that have been linked to human cancer, thus opening up new experimental approaches to study how these genes contribute to malignancy in a genetically tractable experimental model like *Drosophila*.

## 2. Methods

### 2.1. Fly stocks and screen strategy

We used the Gal4/UAS system [17] to drive the expression of both *UAS-l(3)mbt-RNAi* and each of the UAS-RNAi lines to be screened. Gal4 and UAS-RNAi lines were obtained from Bloomington *Drosophila* Stock Center (BDSC) or Vienna *Drosophila* Resource Center (VDRC) [18], with the exception of the *UAS-Tctp-RNAi* line [19]. UAS-RNAi lines inserted in the X chromosome and lines that were not homozygous viable were not used in our screen.

To optimize penetrance and expressivity of the mbt tumour phenotype, we tested the following Gal4 and RNAi lines: *da-Gal4*, *mat4-Gal4*, *Act5C-Gal4*, *Ubi-Gal4* (55851, 7062, 4414 and 32551 from BDSC), and *UAS-l(3)mbt-RNAi* lines (28076 and 35052 from BDSC, and v12709 and v13994 from VDRC). We selected *UAS-l(3)mbt-RNAi* line v12709 and *Ubi-Gal4* for our screen. To further enhance the phenotype, we also introduced *UAS-Dcr2* (24650 from BDSC) in the final stock, which we made heterozygous for the *l(3)mbt^ts1^* allele. In addition, we used a PiggyBac insertion carrying DsRed (Kyoto stock number 140-131) as chromosome marker, a Y-chromosome carrying heat shock-inducible *hid* (Y*^hs-hid^*, 24638 from BDSC), to facilitate the collection of virgins, and a GAL80 expressing balancer (9490 from BDSC) to repress UAS-transgenes expression in the parental flies. The final stock genotype used in the screens is *w^1118^/Y^hs-hid^; Ubi-Gal4, UAS-Dcr2; UAS-l(3)mbt-RNAi, DsRed, l(3)mbt^ts1^/TM6B, tubP-Gal80, Tb.*

Females from this stock were crossed to males carrying each of the transgenic UAS-RNAis to be tested. Eggs collected (0–24 h after egg laying (AEL)) were allowed to develop for up to 9 days (204 ± 12 h AEL), except for control *w^1118^* larvae that were dissected at 5 days AEL. Wandering *Tb^+^* larvae were then selected and brains dissected in PBS. For the candidate screen, images of about 10 larval brains from each cross were taken using a LEICA EC3 camera coupled to a NIKON SMZ800 stereoscope. The images were analysed by a purpose made macro (available upon request) written in ImageJ software [20] to measure maximum brain Feret diameter (henceforth referred to as mbFeret) of the optic lobes pair. Ventral ganglions were either cut out prior to image acquisition or digitally masked before measurement. For the high-content screen, brains were classified upon visual inspection as (1) smaller than wild-type, (2) wild-type, (3) mbt tumour and (4) larger than mbt tumour. UAS-RNAi lines in which three or more brains were smaller than mbt (classes 1 and 2) were tagged as potential suppressors and eventually classified as suppressors if they met the same criteria upon re-screen following the same procedure. In total, 100% (*n* = 22) of the double-blind negative controls using *w^1118^* males instead of males from the UAS-RNAi lines were properly identified.

Other fly strains used in this study were: *UAS-brat-RNAi* (TRiP line, 28590, BDSC), *mei-W68^1^* [21] (4932, BDSC), *Df(2R)BSC782* (27354, BSDC), *l(3)mbt^ts1^* [22] and *brat^k06028^* [23]. Recombinants *brat^k06028^ meiW-68*^1^ were generated by standard genetic techniques. The wild-type strain is *w^1118^.* All crosses were maintained at 29°C except for *brat^k06028^* that were kept at 25°C.

### 2.2. Immunohistochemistry

Immunostaining of whole larval brains was performed as described [24]. Antibodies used in this study include: Rabbit anti-Vasa (1 : 200, Santa Cruz d-260), Rabbit anti-Miranda (1 : 1000) [25] and Mouse anti-Dachshund (Ddac1-1', 1 : 50, DSHB). We used Alexa conjugated secondary antibodies (Life Technologies). DNA was stained with DAPI. Larval brains were mounted in Vectashield (Molecular Probes). Images were acquired with a SP8 Leica confocal microscope and processed in Adobe Photoshop and ImageJ.

### 2.3. Allograft assay

Larval optic lobe and tumour grafts were carried out as described [26].

### 2.4. X-ray treatment

X-ray treatment of larvae was performed by irradiating twice per day from 66–90 h to 143–167 h AEL with a total of 10 Gy per irradiation using a SMART 200 (YXLON International). For X-ray treatment in adults, 58 ± 12 h AEL larval optic lobes were implanted into adult hosts and irradiated as described above. Crosses and injected hosts were maintained at 29°C.

### 2.5. RNA isolation and whole transcriptome amplification

RNA extraction and whole transcriptome amplification (WTA) were performed as described previously [27]. Purity and integrity of the purified RNA was assessed on the Agilent Bioanalyzer 2100 (Agilent Genomics), and RNA concentration was determined with a Nanodrop ND-1000 spectrophotometer (Thermo-Fischer). cDNA Library preparation and amplification were performed following the distributor's (Sigma-Aldrich) recommendations for WTA2. SYBR Green (Sigma-Aldrich) was added to the amplification reaction, which was performed in a real-time PCR instrument to monitor amplification yield.

### 2.6. cDNA labelling and microarrays processing

Amplified cDNA was purified and quantified on a Nanodrop ND-1000 spectrophotometer (Thermo-Fischer). In total, 8 µg cDNA was subsequently fragmented by DNAseI and biotinylated by terminal transferase obtained from GeneChip Mapping 250 K Nsp Assay Kit (Affymetrix). Hybridization, washing, staining and scanning of Affymetrix GeneChip® *Drosophila* Genome 2.0 Array were performed following the manufacturer's recommendations. Scanned images (DAT files) were transformed into intensities (CEL files) by AGCC software (Affymetrix).

### 2.7. Microarray expression profiling

Probeset-based gene expression measurements were generated from Affymetrix image files (‘.CEL’ files) using robust multichip average (RMA) normalization [28]. Genes were selected by setting the Bayesian false discovery rate at 0.05 and requiring an absolute fold change respect to control sample larger than 2.

### 2.8. Statistical analysis

The *p*-value for the difference in ratio of suppressors of mbt tumour growth (mbt-SPRs) among mbt bound genes and non-mbt bound genes was calculated using Pearson's *χ*^2^-test of independence. Other *p*-values were calculated using Mann–Whitney test.

## 3. Results

**Figure 1.**
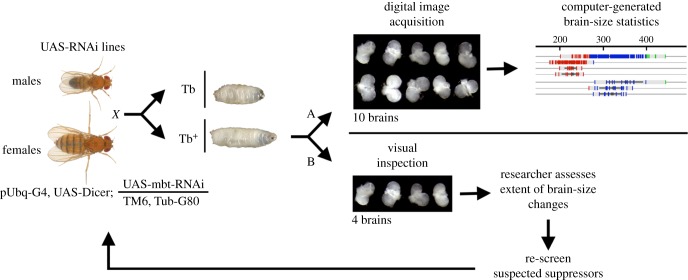
Screen strategy. Females w; *Ubi-Gal4, UAS-Dcr2; UAS-l(3)mbt-RNAi, l(3)mbt^ts1^/TM6B, tubP-Gal80,* are crossed to males carrying each of the transgenic UAS-RNAi lines. Brains from Tb^+^ larvae are dissected and subjected to either (A) an automated classification procedure based on image analyses of low magnification micrographs of about ten brains, or (B) classification based on visual inspection (double blind) of at least four brains and re-screen of suspected suppressors following the same procedure.

We identified mbt-SPRs as UAS-RNAi lines that upon expression from the ubiquitous Ubi-Gal4 promoter significantly reduce *l(3)mbt* larval brain tumour size. The screen was carried out in two steps. We first targeted a selection of 233 candidate genes (electronic supplementary material, table S1) making use of image processing software that classifies the effect of the corresponding RNAi by working out the statistics of mbFerets from a micrograph of dissected brains (figure 1*a* and electronic supplementary material, figure S1). Based on the know-how derived from this first screen, we designed a simpler, faster procedure (figure 1*b*) with which we screened a random selection of 4090 RNAi lines, corresponding to 4075 genes, which represent about 30% of the protein coding sequences in *Drosophila melanogaster* (FlyBase release 6.11) (electronic supplementary material, table S2).

Candidate genes included those that are most highly upregulated in mbt tumours together with others that are functionally related to them [12] and FlyBase. Moreover, given the soma-to-germline transformation that characterizes mbt tumours, we also included 16 *Drosophila* genes predicted by DIOPT [29] to be homologues of human CT genes. Indeed, many of these, like C(3)G/SYCP1 (CT8) and Mei-W68/SPO11 (CT35), are also germline proteins in *Drosophila*, but human TAG (CT49) and Cyclin A1 (CT46) are germline-specific members of protein families with only one *Drosophila* homologue (p24-1 and CycA, respectively), which are expressed ubiquitously.

Out of the 221 RNAi lines that we were able to analyse (12 caused early onset lethality), seven resulted in sizes smaller than wild-type, *37* gave rise to mbt brains that grew to sizes within wild-type range and were therefore classified as mbt-SPRs, 172 had no effect on mbt brain growth and five resulted in brains larger than mbt (electronic supplementary material, table S1 and figure S2).

**Figure 2.**
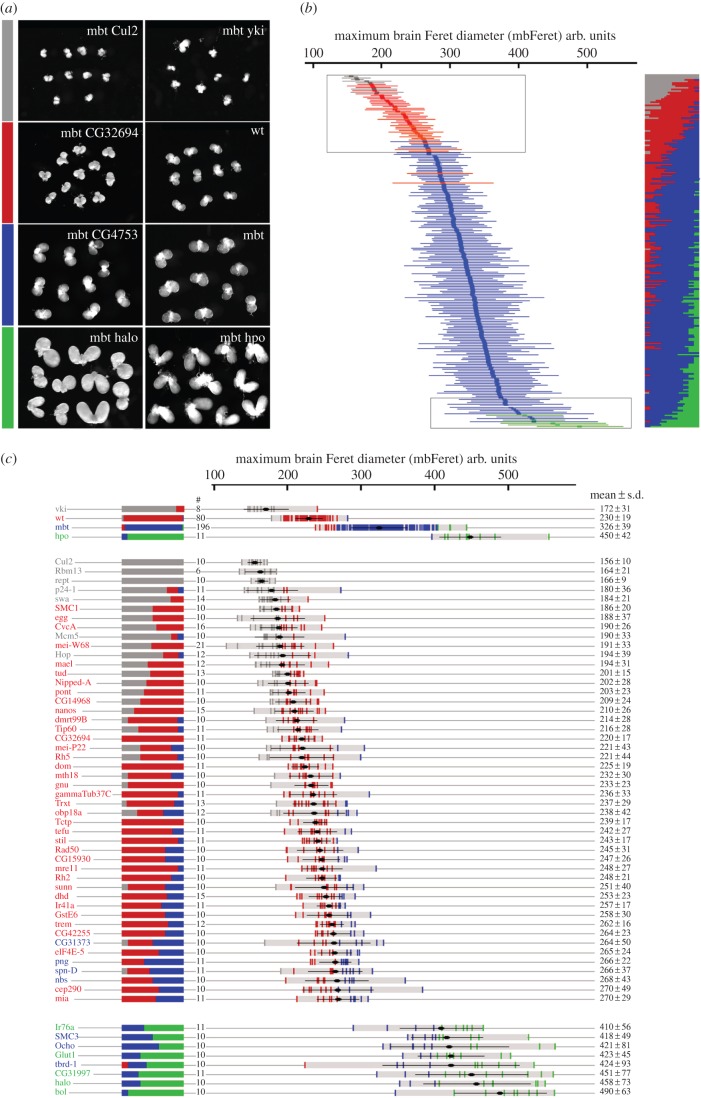
Results from the candidate screen. (*a*) Examples of four larval brain samples found in the screen (left column) alongside brain samples from the four genotypes used to calibrate the algorithm (right column). Colour code indicates brain-size class: smaller than wt (grey), wt size (red), mbt size (blue) and larger than mbt (green). (*b*) Plot showing mbFerets (mean and s.d.) from mbt larvae expressing each of the screened UAS-RNAi lines colour-coded as above. The boxes highlight the regions of the plot that correspond to most of the mbt-SPRs (top) and enhancers (bottom). A condensed plot of colour-coded brain size ranges is shown on the right. (*c*) Detailed plot of mbFerets (mean and s.d.) from control samples and samples of mbt brains expressing the suppressors and enhancers identified in the candidate screen. A condensed plot of brain-size variation and the number of brains in each sample are shown on the left. Actual mean and s.d. values are shown at the right. wt = *w^1118^*.

Figure 2*a* shows four representative examples of larval brains that grew to sizes smaller than wild-type (*mbt cul2; grey*), similar to wild-type (*mbt CG32694; red*), similar to mbt (*mbt CG4753; blue*) and larger than mbt (*mbt halo*; green). Brains from *mbt yorkie* (*yki*), *wild-type*, *mbt* and *mbt hippo* (*hpo*) larvae are also shown as reference. The Salvador–Warts–Hippo (SWH) components *yki* and *hpo* have previously been shown to strongly suppress and enhance mbt growth, respectively [30].

Interestingly, the plot showing the 221 RNAi lines aligned as a function of the mean mbFerets fits to a nearly sigmoidal curve made of three distinct regions defined by their slopes (figure 2*b* and electronic supplementary material, figure S2). The shallow slop at the top of the plot includes 48 RNAis that resulted in mean mbFerets below 270 arb. units (figure 2*b*, upper frame). A detailed view of this part of the plot is shown in figure 2*c*. Most of the RNAis within this region are those that were classified as smaller than wild-type (grey; *n* = 7) or mbt-SPRs (red; *n* = 37). Moreover, all but two of the mbt-SPRs defined by the algorithm are within this section of the plot. These results suggest that mean mbFeret may be a more reliable classifier than mbFeret distribution. This suggestion is further substantiated by the observation that mean mbFerets of the four reference samples used to calibrate the algorithm (*mbt yki*, *wild-type*, *mbt* and *mbt hpo*) are highly significantly different (*p* < 10^−4^). Following this reasoning the four RNAi lines (*CG31373*, *png*, *spn-D* and *nbs*) that were classified as neutral (blue) by the algorithm, but fall within this first part of the plot could also be considered as suppressors of mbt tumour growth.

The second section of the plot, which spans from mbFeret diameters 270–375 arb. units, presents the highest slope (figure 2*b* and electronic supplementary material, figure S2) and correlates tightly with RNAis that were classified as neutral (blue): only two (*Ercc1* and *eIF4E-6*) out of the 159 RNAi lines within this section had been tagged as mbt-SPRs (red). Of note, included in this part of the plot are the *piwi* and *aub* RNAi lines, that unlike the *piwi^1^* and *aub^QC42^* mutant alleles do not efficiently suppress mbt growth [12]. Indeed, false negatives like these are an inherent drawback of any RNAi-based screen. The third and last section of the plot (figure 2*b*, lower frame; electronic supplementary material, figure S2) includes the 14 RNAi lines that resulted in mean mbFerets greater than 376 arb. units. The five RNAi lines at the bottom end of the plot (figure 2*c*) that had been tagged green (enhancers) are *VDRC KK* lines which are likely to activate the SWH pathway [31].

The results of the second, high-content screen are shown in the electronic supplementary material, table S2. Out of the 3609 RNAi lines analysed (481 caused early onset lethality), 113 resulted in larvae with very small brain sizes, in the range of the group classified as smaller than wild-type (grey) in the candidate screen, and 92 behaved as mbt-SPRs (red). The remaining 3404 RNAi lines include those that had no effect on mbt brain size and the relatively few that resulted in enlarged mbt larval brains.

**Figure 3.**
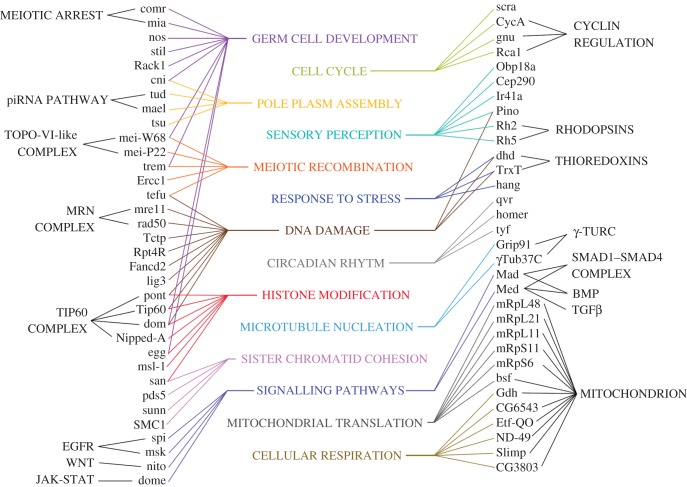
The gene ontology network of mbt-SPRs. Biological functions that are represented by at least two mbt-SPRs are listed in the centre linked to the corresponding mbt-SPRs. Some mbt-SPRs are further linked to molecular complexes, pathways or cellular components.

Altogether we have identified 131 mbt-SPRs. These mbt-SPRs can be linked to different gene ontologies, which include histone modification, DNA damage response (DDR) and germ cell development, among others (electronic supplementary material, table S2 and figure 3).

### 3.1. Upregulation of gene expression is not a good predictor of functional requirement

The results from our candidate screen reveal that 9 of the 12 mbt-SPRs found among the genes that belong to the MBTS are germline-related, even though germline genes represent only a quarter of the MBTS [12]. This result strongly substantiates the functional relevance that acquisition of germline traits has on mbt brain tumour development. Moreover, the much higher discovery rate of mbt-SPRs in the candidate screen than in the high-content screen (16.7%, *n* = 233 versus 2.2%, *n* = 4090; *p*-value < 2.2 × 10^−16^ (*χ*^2^-test of goodness-of-fit) substantiates the criteria for candidate selection.

However, our results also reveal a very poor correlation between unscheduled upregulation and functional requirement. In the candidate screen, 86% (117/136) of the genes that are most highly upregulated in mbt tumours did not behaved as mbt-SPRs. In the high-content screen, only two out of the total 92 (2%) mbt-SPRs identified (*CG11843* and *CG4975*) are significantly upregulated in mbt tumours, the expression levels of 84 mbt-SPRs (82%) are not significantly different in mbt with respect to wild-type brains, and six mbt-SPRs (6%; *Pbgs*, *homer*, *lig3*, *CG1024*, *CG4552* and *CG12182*) are actually downregulated in mbt tumours [12]. Also of relevance is the fact that the rate of mbt-SPR discovery among the genes that have L(3)mbt bound regions (24/862; 2.8%), which are thought to be transcriptionally repressed by the LINT complex in somatic cells [30,32], and the genes that do not (68/3213; 2.1%) are not significantly different (*p* = 0.24). From these observations, we conclude that neither gene upregulation nor the presence of mbt-binding sequences are good predictors of functional requirement.

### 3.2. Ectopic expression of most tumour signature genes is not sufficient to trigger tumorigenesis

**Figure 4.**
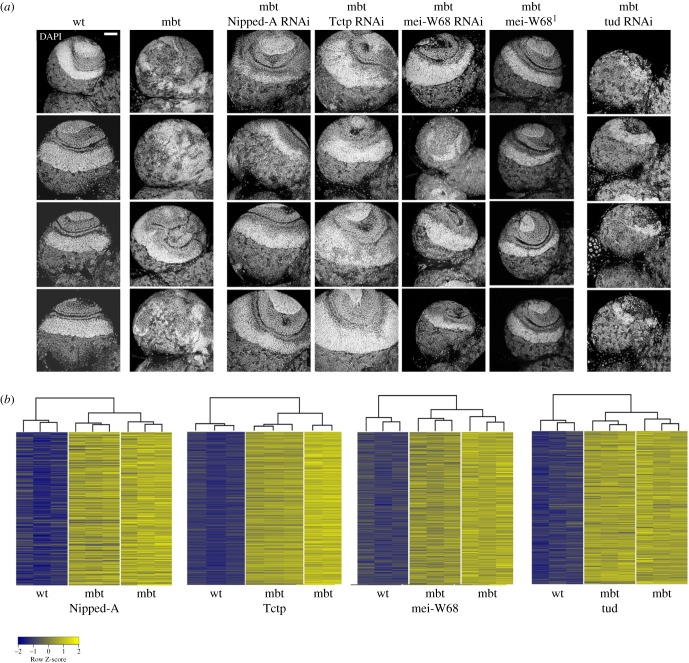
The phenotype of suppressed mbt tumours. (*a*) Array showing confocal ventral sections of four DAPI-stained optic lobes from *w^1118^* (wt), mbt and mbt expressing the suppressors Nipped-A RNAi, Tctp RNAi, mei-W68 RNAi or tud RNAi. Brains from double-mutant larvae *mei-W68^1^; l(3)mbt^ts1^* are also shown. (*b*) Heatmaps of RNA expression profiles of *w^1118^* (wt) brains, mbt brain tumours and mbt tumours suppressed by the expression of Nipped-A RNAi, Tctp RNAi or tud RNAi, as well as in the double-mutant condition *mei-W68^1^; l(3)mbt^ts1^*. Probesets correspond to genes that are significantly upregulated in mbt tumours versus wt larval brains. Expression levels are reported as Row Z-score; blue and yellow indicate low and high expression level, respectively. Dendrograms on the top of the heatmaps show hierarchical clustering between samples. Scale bar, 50 µm.

To get a better understanding of the effect that the mbt-SPRs identified in the screen exert on mbt tumour growth, we examined the gross organization of the central brain, medulla, neuroepithelium and lamina, as revealed by DAPI staining, in brains from l(3)mbt larvae expressing a selection of suppressing RNAi lines (electronic supplementary material, figure S3). We found that penetrance of mbt tumour suppression can be rather variable for any given RNAi line, something that was only to be expected given the wide penetrance range of the mbt tumour phenotype alone. Variable penetrance notwithstanding, we found two major distinguishable phenotypic classes. The largest (greater than 90%) includes brains that, indeed, do not grow beyond wild-type range sizes, but present no visible signs of recovery of wild-type brain anatomy landmarks. This is the case for instance of l(3)mbt brains expressing RNAi lines for *tud, γTub37C*, or *gnu* (electronic supplementary material, figure S3). By contrast, the second class of mbt-SPRs do allow *l(3)mbt* mutant brains to develop such that they very closely resemble wild-type brains (electronic supplementary material, figure S3). Three examples of the second class (*Nipped-A*, *Tctp*, *mei-W68*) and one of the first (*tud*) are shown in figure 4*a*. In the case of *mei-W68*, the recovery of wild-type traits is even more prominent in double-mutant *mei-W68^1^*; *l(3)mbt^ts1^* larvae, which carry the strong loss of function condition *mei-W68^1^* [21]. Interestingly, in contrast with their effect upon mbt tumour growth, the growth of brat tumours is not perturbed at all by depletion of either *tud* or *mei-W68*, thus revealing a certain degree of specificity in the tumour-suppressing effect of these two conditions.

These results open the question of the status of expression of the MBTS genes in these mbt-suppressed conditions that present such a remarkable degree of recovery of wild-type brain lobe development, but are still mutant for mbt. To answer this question, we carried out genome-wide gene expression profiling of mbt tumours suppressed by depletion of *Nipped-A, Tctp* and *mei-W68*, which closely resemble wild-type brains (figure 4*b*). For comparison, we also profiled mbt tumours suppressed by depletion of *tud*, which inhibits mbt tumour growth, but does not rescue wild-type anatomy traits (figure 4*b*).

We found that the majority of the genes that are most upregulated in mbt tumours remain overexpressed in all four tumour-suppressing conditions we assayed (figure 4*b*). Consequently, as far these particular gene probe sets are concerned, hierarchical clustering between samples consistently shows all four tumour-suppressed conditions to be closer to mbt tumours than to wild-type brains. In the cases of mbt tumours suppressed by depletion of *mei-W68, Tctp or Nipped-A,* the percentages are 8.7%, 11.0% and 10.5%, respectively. In the case of mbt tumours suppressed by depletion of *tud* the percentage drops to 1.7%. Interestingly, among the genes downregulated following mbt tumour suppression there are some of the mbt-SPRs identified in our screen. Thus, for instance, the expression levels of *sunn*, *dhd*, *scra* and *l(1)sc* are down in mbt brains depleted for *Nipped-A*; *gnu* and *dmrt99B* are downregulated following depletion of *Tctp*; and *dmrt99B* and *CG42255* are downregulated following depletion of *tud*.

These results demonstrate that upregulation of most of the tumour transcriptome signature genes does not necessarily imply malignant growth.

### 3.3. DNA damage caused by mei-W68 is essential for the growth of *Drosophila* mbt larval brain tumours

*mei-W68* is the *Drosophila* orthologue of *SPO11*, a human cancer/testis gene catalogued as CT35 that is aberrantly expressed in different types of cancer [15]. *SPO11/ mei-W68* encodes a highly conserved trans-esterase that in yeast, plants and animals catalyses the formation of the developmentally programmed double-strand breaks (DSBs) that initiate meiotic recombination [21,33–35].

**Figure 5.**
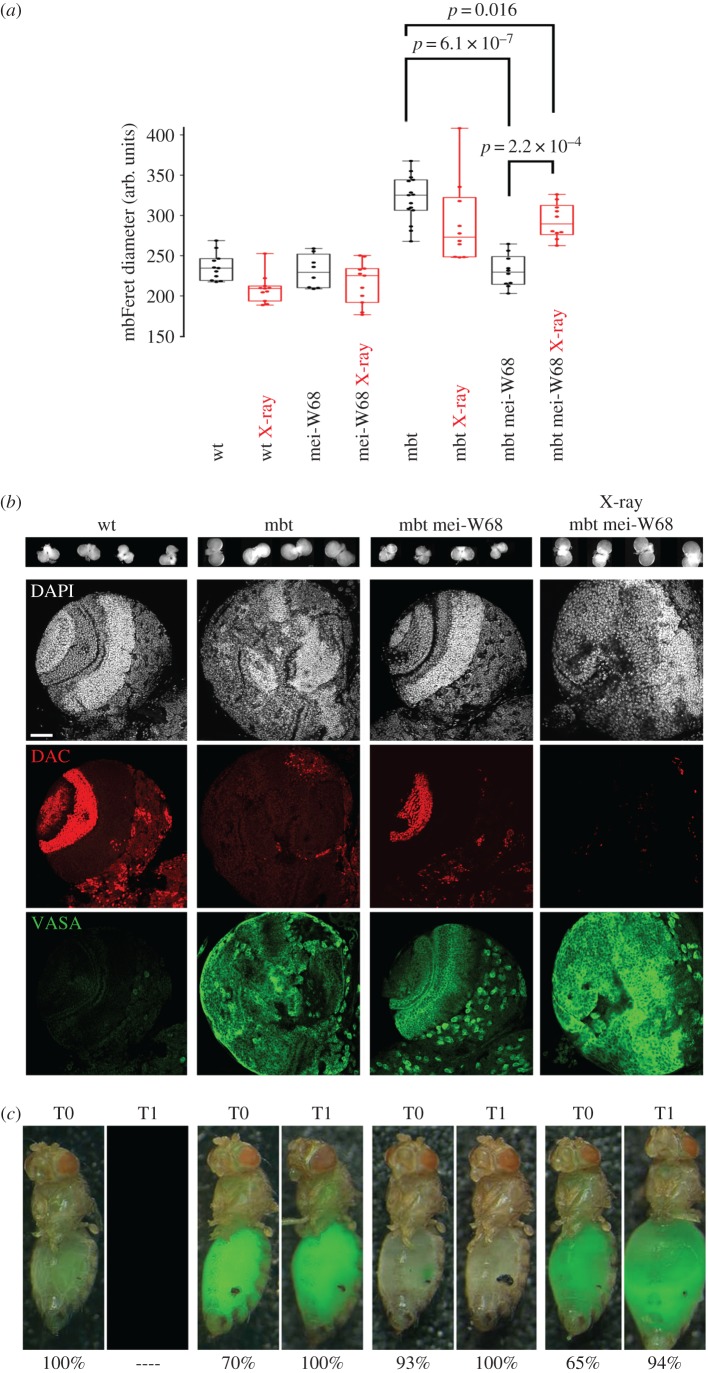
Ionizing radiation can rescue malignant growth in mbt tumours suppressed by depletion of *mei-W68*. (*a*) Box plot of mbFerets from untreated (ctrl, black) or X-irradiated (X-rays, red) wt, mei-W68, mbt and mbt mei-W68 third instar larvae. (*b*) Confocal sections from wt, mbt and mbt mei-W68 (untreated and X-irradiated) larval brains, stained with DAPI (white), anti-DAC (red) and anti-VASA antibodies (green). Untreated mbt mei-W68 double-mutant brains present nearly wild-type anatomy, but resemble mbt tumours following X-ray treatment. VASA expression is upregulated in mbt and mbt mei-W68 larval brains. (*c*) Examples of flies allografted with brain tissue obtained from the corresponding experiment showing the most frequent phenotype. Green signal corresponds to GFP-labelled tumour tissue. wt = *w^1118^*, mbt = *l(3)mbt^ts1^,* mei-W68 *=* *mei-W68^1^.* Scale bar, 50 µm.

Elegant experiments carried out in *Saccharomyces cerevisiae*, *Caenorhabditis elegans* and *D. melanogaster* demonstrate that the absence of developmentally programmed DSB, and the concomitant lack of meiotic recombination brought about by *mei-W68/SPO11* loss of function can be alleviated by DNA damage caused by X-irradiation [35–37]. Following the same reasoning, we investigated whether X-irradiation could rescue tumour growth in mbt mutant larvae depleted for *mei-W68*. To this end, we subjected larvae to a dosage of 10 Gy, delivered twice daily, for a period of 5 days, starting at late 2nd instar (electronic supplementary material, figure S5*a*). Consistent with the detrimental effect of ionizing radiation on cell survival, irradiated *wild-type*, *mei-W68* and *l(3)mbt* larvae presented slightly smaller brains sizes than the corresponding non-irradiated controls (figure 5*a* and electronic supplementary material, figure S6). However, brains from X-irradiated double-mutant *mei-W68^1^*; *l(3)mbt^ts1^* larvae grew significantly larger (*p* = 2.2 × 10^−4^) than those from untreated larvae of the same genotype, up to the size range of X-irradiated mbt larval brain tumours (figure 5*a*).

Moreover, while X-irradiation did not have any gross effect on brain anatomy (as revealed by DAPI and anti-DAC staining) in *wild-type*, *l(3)mbt* or *mei-W68* larvae (electronic supplementary material, figure S6), it did notably affect *mei-W68*; *l(3)mbt* double-mutant brains (figure 5*b*). In the absence of X-irradiation, as shown before, the organization of neuroepithelium, lamina and medulla in *mei-W68*; *l(3)mbt* double-mutant brains is closely similar to that of control *wild-type* larvae (figure 5*b*). The abundant VASA expression reveals the double-mutant genotype of the otherwise phenotypically wild-type *mei-W68*; *l(3)mbt* brains (figure 5*b*). Upon X-irradiation, however, *mei-W68*; *l(3)mbt* brains loose wild-type traits and closely resemble mbt tumours (figure 5*b*).

X-irradiated *mei-W68^1^*; *l(3)mbt^ts1^* brains developed as malignant tumours at rates of 65% upon implantation in adult hosts (T0), and 94% at the first re-implantation (T1), which are indistinguishable from those of non-irradiated mbt brain tumours (70% and 100%, respectively; figure 5*c*). By sharp contrast, most (93%; *n* = 45) implants of untreated, *mei-W68^1^*; *l(3)mbt^ts1^* double-mutant brains did not grow upon allograft (figure 5*c*) and the remaining 7% presented some growth that nonetheless arrested well before filling the abdominal cavity and failed to continue upon re-implantation (figure 5*c*). An alternative protocol in which the same course of X-irradiation is delivered to adult hosts implanted with brains from 2nd instar larvae, as described in the electronic supplementary material, figure S5*b*, gave identical results.

These results demonstrate that X-irradiation can complement the suppressing effect that loss of mei-W68 function has on mbt tumour growth, hence strongly suggesting that mei-W68 contributes to mbt tumour development by causing DNA damage. Allografted X-rayed *mei-W68*; *l(3)mbt* double-mutant tissue continues to grow for many rounds of implantation, months after the last exposure to X-rays. This result may be taken to suggest that the pro-tumoural role of DNA damage might be limited to early stages of tumorigenesis and be dispensable for mbt tumour growth at later times. However, we cannot discard that once mbt tumours are established, alternative mechanisms generate a basal state of DNA damage that no longer depends on mei-W68 function.

## 4. Discussion

Larval brain neoplasms induced by the loss of *l(3)mbt* present the key traits of malignant growth [1,4,13]. They also recapitulate some features of specific human cancers like upregulation of cancer/testis (CT) genes [12,15] and the sensitivity to inhibition of the K^+^ channel *eag/EAG2*, which also affects human medulloblastomas [38,39]. Moreover, *L3MBTL3* is deleted in some human medulloblastoma cell lines whose malignant phenotypes become attenuated upon re-expression of *L3MBTL3* [40].

We have combined the *Drosophila l(3)mbt* experimental brain tumour model and well-established RNAi technology methods and resources to interrogate the genome for biological processes that can be targeted to inhibit malignant growth. To select for functions whose depletion has a much stronger effect on the tumour than in the other tissues of the tumour-bearing animal, we chose to drive RNAi expression ubiquitously, hence filtering out RNAis that cause lethality or severely impair development. There are two types of functions that can be expected to meet these criteria: those that are under higher demand in the tumour than in the rest of the body and those that are tumour-specific. We have found both kinds in our screen.

Most of the genes that we have identified belong to the first kind; they are required for normal development and some of them cause lethality in lack-of-function conditions. Examples of these are mitochondrial proteins involved in oxidative phosphorylation and respiration like Etf-QO, Gdh, ND-49 and Slimp [41–43], Bsf, which controls mitochondrial translation [44], and a few mitochondrial ribosomal proteins. Interestingly, loss of Slimp, like loss of L(3)MBT, overcomes the growth arrest caused by loss of E2F [45]. These findings suggest that mbt tumours might be more sensitive than wild-type tissues to loss of mitochondria-related functions. Finding this kind of suppressor in our screen reveals that for the corresponding genes a function threshold must exist at which normal development can proceed, but tumour growth is severely curtailed. The protein products of these genes might be well-suited targets for pharmacological inhibition.

Even better suited candidates for pharmacological inhibition are the proteins encoded by genes that belong to the second kind; their role in normal development is minimal so that individuals carrying lack-of-function conditions can reach adulthood, yet they are essential for mbt tumour growth. Examples of this kind are *TrxT, dhd*, *mael* and *mei-W68* whose functions are tightly linked to the germline [21,46–51]. The thioredoxins TrxT and Dhd, both members of the mbt tumour signature [12], are specific of the male and female germline, respectively [46,47]. Thioredoxins are highly conserved anti-oxidant proteins that play a critical role in the regulation of intracellular redox homeostasis. The role of ROS in cancer cells is extremely complex [52]. Together with solid evidence substantiating the potential tumorigenic effect of an oxidative environment, there is also evidence showing that high levels of reduced glutathione caused by treatment with common antioxidants increases the migration and invasive properties of human malignant melanoma cells [53] and that the thioredoxin pathway is important for the survival of cancer cells [54].

Mael and Mei-W68 are orthologues of human cancer testis CT128 (MAEL) and CT35 (SPO11), respectively. The finding of Mei-W68/SPO11, the endonuclease that forms DSBs during meiosis, as an efficient suppressor of mbt tumour growth provides the first experimental evidence for the long suspected pro-tumoural role of *mei-W68/SPO11*. Like *mei-W68/SPO11*, other mbt-SPRs found in our screen correspond to genes that normally function during meiotic recombination. Upregulation of meiotic proteins is not uncommon in human somatic tumours where they are suspected to perturb the mitotic cycle, hence contributing to genome instability, a distinct feature of many types of cancer [15,55–57]. Our data demonstrating that ionizing radiation can functionally substitute for *Drosophila* Mei-W68 strongly suggests that the pro-tumoural role of Mei-W68/SPO11 is associated with its endonuclease activity.

Several mbt-SPRs encode proteins involved in DDR. Among them are *telomere fusion* (*tefu),* the *Drosophila* orthologue of human ATM (Ataxia Telangiectasia Mutated); *meiotic recombination 11* (*mre11*) and *rad50*, members of the MRN complex that activates ATM; and *Nipped-A*, *pontin* (*pont*), *Tip60* and *domino* (*dom*), all of them components of the Tip60 complex, which is also a mandatory activator of ATM [58]. Activated ATM phosphorylates many downstream target proteins, one of which is H2Av [59], the *Drosophila* homologue of human Histone 2AX [60] that upon phosphorylation works as a platform to recruit various DNA repair proteins, histone modifiers and chromatin remodellers [61,62]. Tip60 and Domino/p400 catalyse the exchange of phospho-H2Av with non-phosphorylated H2Av [61]. Also *TrxT* and *dhd* belong to the ‘MMS (methyl methanesulfonate) survival network’ of genes that mediate the cellular response to DNA damage [63]. It is conceivable that functions involved in the DDR axis might become essential for the survival of tumours like mbt, which depends upon DNA endonuclease activity, and therefore depletion of these genes may result in synthetic lethality. However, the situation may be far more complex because most of these proteins have functions outside the DDR axis. In *Drosophila*, ATM also works on telomere position effect [64] and alternative splicing [65] and various components of the Tip60 complex control several signalling pathways and have effects on transcriptional activation, cell cycle progression, stem cell maintenance and differentiation [66–68].

Also related to repairing DNA damage is the mbt-SPR *Tctp.* In *Drosophila*, TCTP binds and regulates ATM and interacts genetically with each component of the MRN complex as well as with other genes involved in DNA repair [69]. Interestingly, TCTP is specifically required for DSB repair in an ATM-dependent manner but not for single-strand breaks repair [69,70]. The finding that depletion of TCTP allows *l(3)mbt* larvae to develop phenotypically normal brains is particularly striking because, in humans, TCTP downregulation is suspected to play a key role in the rare cases of tumour reversion [29,71,72].

An interesting conclusion to be drawn from our results is that gene upregulation is not a good predictor of functional requirement: most (91%; 84/92) of the mbt-SPRs identified in the high-content screen correspond to genes that are not significantly upregulated in mbt tumours and 6% correspond to genes that are actually significantly downregulated in mbt tumours. The presence of L(3)mbt bound regions [30] is not a good predictor either because the fraction of mbt-SPRs among genes that have such sequences is not significantly different from the fraction of mbt-SPRs among the genes that do not. Moreover, we have found that the majority of the genes that are ectopically upregulated in mbt tumours remain so in *l(3)mbt* larval brains in which tumour growth has been suppressed by depletion of *Tctp*, *Nipped-A* or *mei-W68*. The fact that mbt tumour suppression and the consequent rescue of wild-type morphology traits can take place in brains with a gene expression landscape that is rather close to that of mbt tumours demonstrates that ectopic expression of dozens of genes, including many germline genes, is not on its own necessarily tumorigenic.

## 5. Conclusion

We have identified a collection of targets that inhibit malignant neoplasm growth in *Drosophila* and may serve as potential leads for the development of new therapeutic strategies. Moreover, the finding that homologues of genes like *TCTP* and *SPO11*, which are the subject of intense research in the context of human oncology, have a key role in *Drosophila* neoplasms substantiates the potential of this genetically tractable organism as a model to elucidate the molecular mechanisms of malignant growth.

## Supplementary Material

Supplemental Figures and figure legends 1 to 6

## Supplementary Material

Table S1. Results from the candidate screen.

## Supplementary Material

Table S2. Results from the high-content screen.

## Data accessibility

The datasets supporting this article have been uploaded as part of the electronic supplementary material.

## References

[1] Caussinus E, Gonzalez C 2005 Induction of tumor growth by altered stem-cell asymmetric division in *Drosophila melanogaster*. Nat. Genet. 37, 1125–1129. (doi:10.1038/ng1632) 16142234 10.1038/ng1632

[2] Gateff E 1978 Malignant neoplasms of genetic origin in *Drosophila melanogaster*. Science 200, 1448–1459. (doi:10.1126/science.96525) 96525 10.1126/science.96525

[3] Rudrapatna VA, Cagan RL, Das TK 2012 *Drosophila* cancer models. Dev. Dyn. 241, 107–118. (doi:10.1002/dvdy.22771) 22038952 10.1002/dvdy.22771 PMC3677821

[4] Gonzalez C 2013 *Drosophila melanogaster*: a model and a tool to investigate malignancy and identify new therapeutics. Nat. Rev. Cancer 13, 172–183. (doi:10.1038/nrc3461) 23388617 10.1038/nrc3461

[5] Figueroa-Clarevega A, Bilder D 2015 Malignant *Drosophila* tumors interrupt insulin signaling to induce cachexia-like wasting. Dev. Cell 33, 47–55. (doi:10.1016/j.devcel.2015.03.001) 25850672 10.1016/j.devcel.2015.03.001 PMC4390765

[6] Kwon Y, Song W, Droujinine IA, Hu Y, Asara JM, Perrimon N 2015 Systemic organ wasting induced by localized expression of the secreted insulin/IGF antagonist ImpL2. Dev. Cell 33, 36–46. (doi:10.1016/j.devcel.2015.02.012) 25850671 10.1016/j.devcel.2015.02.012 PMC4437243

[7] Markstein M, Dettorre S, Cho J, Neumuller RA, Craig-Muller S, Perrimon N 2014 Systematic screen of chemotherapeutics in *Drosophila* stem cell tumors. Proc. Natl Acad. Sci. USA 111, 4530–4535. (doi:10.1073/pnas.1401160111) 24616500 10.1073/pnas.1401160111 PMC3970492

[8] Willoughby LF, Schlosser T, Manning SA, Parisot JP, Street IP, Richardson HE, Humbert PO, Brumby AM 2013 An *in vivo* large-scale chemical screening platform using *Drosophila* for anti-cancer drug discovery. Dis. Model Mech. 6, 521–529. (doi:10.1242/dmm.009985) 22996645 10.1242/dmm.009985 PMC3597034

[9] Dar AC, Das TK, Shokat KM, Cagan RL 2012 Chemical genetic discovery of targets and anti-targets for cancer polypharmacology. Nature 486, 80–84. (doi:10.1038/nature11127) 22678283 10.1038/nature11127 PMC3703503

[10] Parsons LM, Grzeschik NA, Amaratunga K, Burke P, Quinn LM, Richardson HE 2017 A kinome RNAi screen in *Drosophila* identifies novel genes interacting with Lgl, aPKC and Crb cell polarity genes in epithelial tissues. G3 (Bethesda) 7, 2497–2509. (doi:10.1534/g3.117.043513) 28611255 10.1534/g3.117.043513 PMC5555457

[11] Anderson AM, Bailetti AA, Rodkin E, De A, Bach EA 2017 A genetic screen reveals an unexpected role for yorkie signaling in JAK/STAT-dependent hematopoietic malignancies in *Drosophila melanogaster*. G3 (Bethesda) 7, 2427–2438. (doi:10.1534/g3.117.044172) 28620086 10.1534/g3.117.044172 PMC5555452

[12] Janic A, Mendizabal L, Llamazares S, Rossell D, Gonzalez C 2010 Ectopic expression of germline genes drives malignant brain tumor growth in *Drosophila*. Science 330, 1824–1827. (doi:10.1126/science.1195481) 21205669 10.1126/science.1195481

[13] Gateff E, Loffler T, Wismar J 1993 A temperature-sensitive brain tumor suppressor mutation of *Drosophila melanogaster*: developmental studies and molecular localization of the gene. Mech. Dev. 41, 15–31. (doi:10.1016/0925-4773(93)90052-Y) 8507589 10.1016/0925-4773(93)90052-y

[14] Almeida LG et al. 2009 CTdatabase: a knowledge-base of high-throughput and curated data on cancer-testis antigens. Nucleic. Acids Res. 37, D816–D819. (doi:10.1093/nar/gkn673) 18838390 10.1093/nar/gkn673 PMC2686577

[15] Simpson AJ, Caballero OL, Jungbluth A, Chen YT, Old LJ 2005 Cancer/testis antigens, gametogenesis and cancer. Nat. Rev. Cancer 5, 615–625. (doi:10.1038/nrc1669) 16034368 10.1038/nrc1669

[16] Feichtinger J, Larcombe L, McFarlane RJ 2014 Meta-analysis of expression of l(3)mbt tumor-associated germline genes supports the model that a soma-to-germline transition is a hallmark of human cancers. Int. J. Cancer 134, 2359–2365. (doi:10.1002/ijc.28577) 24243547 10.1002/ijc.28577 PMC4166677

[17] Brand AH, Manoukian AS, Perrimon N 1994 Ectopic expression in *Drosophila*. Methods Cell Biol. 44, 635–654. (doi:10.1016/S0091-679X(08)60936-X) 7707973 10.1016/s0091-679x(08)60936-x

[18] Dietzl G et al. 2007 A genome-wide transgenic RNAi library for conditional gene inactivation in *Drosophila*. Nature 448, 151–156. (doi:10.1038/nature05954) 17625558 10.1038/nature05954

[19] Hsu YC, Chern JJ, Cai Y, Liu M, Choi KW 2007 *Drosophila* TCTP is essential for growth and proliferation through regulation of dRheb GTPase. Nature 445, 785–788. (doi:10.1038/nature05528) 17301792 10.1038/nature05528

[20] Schneider CA, Rasband WS, Eliceiri KW 2012 NIH Image to ImageJ: 25 years of image analysis. Nat. Methods 9, 671–675. (doi:10.1038/nmeth.2089) 22930834 10.1038/nmeth.2089 PMC5554542

[21] McKim KS, Hayashi-Hagihara A 1998 mei-W68 in *Drosophila melanogaster* encodes a Spo11 homolog: evidence that the mechanism for initiating meiotic recombination is conserved. Genes. Dev. 12, 2932–2942. (doi:10.1101/gad.12.18.2932) 9744869 10.1101/gad.12.18.2932 PMC317166

[22] Yohn CB, Pusateri L, Barbosa V, Lehmann R 2003 l(3)malignant brain tumor and three novel genes are required for *Drosophila* germ-cell formation. Genetics 165, 1889–1900. 14704174 10.1093/genetics/165.4.1889 PMC1462896

[23] Spradling AC, Stern D, Beaton A, Rhem EJ, Laverty T, Mozden N, Misra S, Rubin GM 1999 The Berkeley *Drosophila* Genome Project gene disruption project: single P-element insertions mutating 25% of vital *Drosophila* genes. Genetics 153, 135–177. 10471706 10.1093/genetics/153.1.135 PMC1460730

[24] Gonzalez C, Glover DM 1993 Techniques for studying mitosis in *Drosophila*. In The cell cycle (eds Fantes P, Brooks R), pp. 143–175. Oxford, UK: Oxford University Press.

[25] Mollinari C, Lange B, Gonzalez C 2002 Miranda, a protein involved in neuroblast asymmetric division, is associated with embryonic centrosomes of *Drosophila melanogaster*. Biol. Cell 94, 1–13. (doi:10.1016/S0248-4900(02)01181-4) 12000142 10.1016/s0248-4900(02)01181-4

[26] Rossi F, Gonzalez C 2015 Studying tumor growth in *Drosophila* using the tissue allograft method. Nat. Protoc. 10, 1525–1534. (doi:10.1038/nprot.2015.096) 26357008 10.1038/nprot.2015.096

[27] Gonzalez-Roca E, Garcia-Albeniz X, Rodriguez-Mulero S, Gomis RR, Kornacker K, Auer H 2010 Accurate expression profiling of very small cell populations. PLoS ONE 5, e14418 (doi:10.1371/journal.pone.0014418) 21203435 10.1371/journal.pone.0014418 PMC3010985

[28] Irizarry RA, Hobbs B, Collin F, Beazer-Barclay YD, Antonellis KJ, Scherf U, Speed TP 2003 Exploration, normalization, and summaries of high density oligonucleotide array probe level data. Biostatistics 4, 249–264. (doi:10.1093/biostatistics/4.2.249) 12925520 10.1093/biostatistics/4.2.249

[29] Amson R et al. 2012 Reciprocal repression between P53 and TCTP. Nat. Med. 18, 91–99. (doi:10.1038/nm.2546) 10.1038/nm.2546 22157679

[30] Richter C, Oktaba K, Steinmann J, Muller J, Knoblich JA 2011 The tumour suppressor L(3)mbt inhibits neuroepithelial proliferation and acts on insulator elements. Nat. Cell Biol. 13, 1029–1039. (doi:10.1038/ncb2306) 21857667 10.1038/ncb2306 PMC3173870

[31] Vissers JH, Manning SA, Kulkarni A, Harvey KF 2016 A *Drosophila* RNAi library modulates Hippo pathway-dependent tissue growth. Nat. Commun. 7, 10368 (doi:10.1038/ncomms10368) 26758424 10.1038/ncomms10368 PMC4735554

[32] Meier K, Mathieu EL, Finkernagel F, Reuter LM, Scharfe M, Doehlemann G, Jarek M, Brehm A 2012 LINT, a novel dL(3)mbt-containing complex, represses malignant brain tumour signature genes. PLoS Genet. 8, e1002676 (doi:10.1371/journal.pgen.1002676) 22570633 10.1371/journal.pgen.1002676 PMC3342951

[33] Robert T, Nore A, Brun C, Maffre C, Crimi B, Bourbon HM, de Massy B 2016 The TopoVIB-Like protein family is required for meiotic DNA double-strand break formation. Science 351, 943–949. (doi:10.1126/science.aad5309) 26917764 10.1126/science.aad5309

[34] Liu H, Jang JK, Kato N, McKim KS 2002 mei-P22 encodes a chromosome-associated protein required for the initiation of meiotic recombination in *Drosophila melanogaster*. Genetics 162, 245–258. 12242237 10.1093/genetics/162.1.245 PMC1462256

[35] Lake CM, Nielsen RJ, Hawley RS 2011 The *Drosophila* zinc finger protein trade embargo is required for double strand break formation in meiosis. PLoS Genet. 7, e1002005 (doi:10.1371/journal.pgen.1002005) 21383963 10.1371/journal.pgen.1002005 PMC3044681

[36] Thorne LW, Byers B 1993 Stage-specific effects of X-irradiation on yeast meiosis. Genetics 134, 29–42. 8514137 10.1093/genetics/134.1.29 PMC1205431

[37] Dernburg AF, McDonald K, Moulder G, Barstead R, Dresser M, Villeneuve AM 1998 Meiotic recombination in *C. elegans* initiates by a conserved mechanism and is dispensable for homologous chromosome synapsis. Cell 94, 387–398. (doi:10.1016/S0092-8674(00)81481-6) 9708740 10.1016/s0092-8674(00)81481-6

[38] Pardo LA, del Camino D, Sanchez A, Alves F, Bruggemann A, Beckh S, Stuhmer W 1999 Oncogenic potential of EAG K^+^ channels. EMBO J. 18, 5540–5547. (doi:10.1093/emboj/18.20.5540) 10523298 10.1093/emboj/18.20.5540 PMC1171622

[39] Huang X et al. 2015 EAG2 potassium channel with evolutionarily conserved function as a brain tumor target. Nat. Neurosci. 18, 1236–1246. (doi:10.1038/nn.4088) 26258683 10.1038/nn.4088 PMC4639927

[40] Northcott PA et al. 2009 Multiple recurrent genetic events converge on control of histone lysine methylation in medulloblastoma. Nat. Genet. 41, 465–472. (doi:10.1038/ng.336) 19270706 10.1038/ng.336 PMC4454371

[41] Watmough NJ, Frerman FE 2010 The electron transfer flavoprotein: ubiquinone oxidoreductases. Biochim. Biophys. Acta 1797, 1910–1916. (doi:10.1016/j.bbabio.2010.10.007) 20937244 10.1016/j.bbabio.2010.10.007

[42] Tripoli G, D'Elia D, Barsanti P, Caggese C 2005 Comparison of the oxidative phosphorylation (OXPHOS) nuclear genes in the genomes of *Drosophila melanogaster*, *Drosophila pseudoobscura* and *Anopheles gambiae*. Genome. Biol. 6, R11 (doi:10.1186/gb-2005-6-2-r11) 15693940 10.1186/gb-2005-6-2-r11 PMC551531

[43] Guitart T, Leon Bernardo T, Sagales J, Stratmann T, Bernues J, Ribas de Pouplana L 2010 New aminoacyl-tRNA synthetase-like protein in Insecta with an essential mitochondrial function. J. Biol. Chem. 285, 38 157–38 166. (doi:10.1074/jbc.M110.167486) 10.1074/jbc.M110.167486 PMC2992249 20870726

[44] Bratic A et al. 2011 The bicoid stability factor controls polyadenylation and expression of specific mitochondrial mRNAs in *Drosophila melanogaster*. PLoS Genet. 7, e1002324 (doi:10.1371/journal.pgen.1002324) 22022283 10.1371/journal.pgen.1002324 PMC3192837

[45] Ambrus AM, Rasheva VI, Nicolay BN, Frolov MV 2009 Mosaic genetic screen for suppressors of the de2f1 mutant phenotype in *Drosophila*. Genetics 183, 79–92. (doi:10.1534/genetics.109.104661) 19546319 10.1534/genetics.109.104661 PMC2746169

[46] Salz HK, Flickinger TW, Mittendorf E, Pellicena-Palle A, Petschek JP, Albrecht EB 1994 The *Drosophila* maternal effect locus deadhead encodes a thioredoxin homolog required for female meiosis and early embryonic development. Genetics 136, 1075–1086. 7516301 10.1093/genetics/136.3.1075 PMC1205864

[47] Svensson MJ, Chen JD, Pirrotta V, Larsson J 2003 The *ThioredoxinT* and *deadhead* gene pair encode testis- and ovary-specific thioredoxins in *Drosophila* melanogaster. Chromosoma 112, 133–143. (doi:10.1007/s00412-003-0253-5) 14579129 10.1007/s00412-003-0253-5

[48] Findley SD, Tamanaha M, Clegg NJ, Ruohola-Baker H 2003 *Maelstrom*, a *Drosophila* spindle-class gene, encodes a protein that colocalizes with Vasa and RDE1/AGO1 homolog, Aubergine, in nuage. Development 130, 859–871. (doi:10.1242/dev.00310) 12538514 10.1242/dev.00310

[49] Pennetta G, Pauli D 1997 *stand still*, a *Drosophila* gene involved in the female germline for proper survival, sex determination and differentiation. Genetics 145, 975–987. 9093851 10.1093/genetics/145.4.975 PMC1207901

[50] Thomson T, Lasko P 2004 *Drosophila tudor* is essential for polar granule assembly and pole cell specification, but not for posterior patterning. Genesis 40, 164–170. (doi:10.1002/gene.20079) 15495201 10.1002/gene.20079

[51] Krishnan B, Thomas SE, Yan R, Yamada H, Zhulin IB, McKee BD 2014 Sisters unbound is required for meiotic centromeric cohesion in *Drosophila melanogaster*. Genetics 198, 947–965. (doi:10.1534/genetics.114.166009) 25194162 10.1534/genetics.114.166009 PMC4224182

[52] Tong L, Chuang CC, Wu S, Zuo L 2015 Reactive oxygen species in redox cancer therapy. Cancer Lett. 367, 18–25. (doi:10.1016/j.canlet.2015.07.008) 26187782 10.1016/j.canlet.2015.07.008

[53] Gal K Le et al. 2015 Antioxidants can increase melanoma metastasis in mice. Sci. Transl. Med. 7, 308re308 (doi:10.1126/scitranslmed.aad3740) 10.1126/scitranslmed.aad3740 26446958

[54] Harris IS et al. 2015 Glutathione and thioredoxin antioxidant pathways synergize to drive cancer initiation and progression. Cancer Cell 27, 211–222. (doi:10.1016/j.ccell.2014.11.019) 25620030 10.1016/j.ccell.2014.11.019

[55] Nielsen AY, Gjerstorff MF 2016 Ectopic expression of testis germ cell proteins in cancer and its potential role in genomic instability. Int. J. Mol. Sci. 17, 890 (doi:10.3390/ijms17060890) 10.3390/ijms17060890 PMC4926424 27275820

[56] Lindsey SF, Byrnes DM, Eller MS, Rosa AM, Dabas N, Escandon J, Grichnik JM 2013 Potential role of meiosis proteins in melanoma chromosomal instability. J. Skin Cancer 2013, 190109 (doi:10.1155/2013/190109) 23840955 10.1155/2013/190109 PMC3694528

[57] Vogelstein B, Papadopoulos N, Velculescu VE, Zhou S, Diaz LA Jr, Kinzler KW 2013 Cancer genome landscapes. Science 339, 1546–1558. (doi:10.1126/science.1235122) 23539594 10.1126/science.1235122 PMC3749880

[58] Paull TT 2015 Mechanisms of ATM activation. Annu. Rev. Biochem. 84, 711–738. (doi:10.1146/annurev-biochem-060614-034335) 25580527 10.1146/annurev-biochem-060614-034335

[59] Joyce EF, Pedersen M, Tiong S, White-Brown SK, Paul A, Campbell SD, McKim KS 2011 *Drosophila* ATM and ATR have distinct activities in the regulation of meiotic DNA damage and repair. J. Cell Biol. 195, 359–367. (doi:10.1083/jcb.201104121) 22024169 10.1083/jcb.201104121 PMC3206348

[60] Madigan JP, Chotkowski HL, Glaser RL 2002 DNA double-strand break-induced phosphorylation of *Drosophila* histone variant H2Av helps prevent radiation-induced apoptosis. Nucleic. Acids Res. 30, 3698–3705. (doi:10.1093/nar/gkf496) 12202754 10.1093/nar/gkf496 PMC137418

[61] Kusch T, Florens L, Macdonald WH, Swanson SK, Glaser RL, Yates JR III, Abmayr SM, Washburn MP, Workman JL 2004 Acetylation by Tip60 is required for selective histone variant exchange at DNA lesions. Science 306, 2084–2087. (doi:10.1126/science.1103455) 15528408 10.1126/science.1103455

[62] Turinetto V, Giachino C 2015 Multiple facets of histone variant H2AX: a DNA double-strand-break marker with several biological functions. Nucleic. Acids Res. 43, 2489–2498. (doi:10.1093/nar/gkv061) 25712102 10.1093/nar/gkv061 PMC4357700

[63] Ravi D, Wiles AM, Bhavani S, Ruan J, Leder P, Bishop AJ 2009 A network of conserved damage survival pathways revealed by a genomic RNAi screen. PLoS Genet. 5, e1000527 (doi:10.1371/journal.pgen.1000527) 19543366 10.1371/journal.pgen.1000527 PMC2688755

[64] Oikemus SR, McGinnis N, Queiroz-Machado J, Tukachinsky H, Takada S, Sunkel CE, Brodsky MH 2004 *Drosophila* atm/telomere fusion is required for telomeric localization of HP1 and telomere position effect. Genes. Dev. 18, 1850–1861. (doi:10.1101/gad.1202504) 15256487 10.1101/gad.1202504 PMC517405

[65] Katzenberger RJ, Marengo MS, Wassarman DA 2006 ATM and ATR pathways signal alternative splicing of *Drosophila* TAF1 pre-mRNA in response to DNA damage. Mol. Cell Biol. 26, 9256–9267. (doi:10.1128/MCB.01125-06) 17030624 10.1128/MCB.01125-06 PMC1698527

[66] Gause M, Eissenberg JC, Macrae AF, Dorsett M, Misulovin Z, Dorsett D 2006 Nipped-A, the Tra1/TRRAP subunit of the *Drosophila* SAGA and Tip60 complexes, has multiple roles in Notch signaling during wing development. Mol. Cell Biol. 26, 2347–2359. (doi:10.1128/MCB.26.6.2347-2359.2006) 16508010 10.1128/MCB.26.6.2347-2359.2006 PMC1430305

[67] Bellosta P, Hulf T, Balla Diop S, Usseglio F, Pradel J, Aragnol D, Gallant P 2005 Myc interacts genetically with Tip48/Reptin and Tip49/Pontin to control growth and proliferation during *Drosophila* development. Proc. Natl Acad. Sci. USA 102, 11 799–11 804. (doi:10.1073/pnas.0408945102) 10.1073/pnas.0408945102 PMC1187951 16087886

[68] Bauer A, Chauvet S, Huber O, Usseglio F, Rothbacher U, Aragnol D, Kemler R, Pradel J 2000 Pontin52 and reptin52 function as antagonistic regulators of beta-catenin signalling activity. EMBO J. 19, 6121–6130. (doi:10.1093/emboj/19.22.6121) 11080158 10.1093/emboj/19.22.6121 PMC305835

[69] Hong ST, Choi KW 2013 TCTP directly regulates ATM activity to control genome stability and organ development in *Drosophila melanogaster*. Nat. Commun. 4, 2986 (doi:10.1038/ncomms3986) 24352200 10.1038/ncomms3986

[70] Zhang J, de Toledo SM, Pandey BN, Guo G, Pain D, Li H, Azzam EI 2012 Role of the translationally controlled tumor protein in DNA damage sensing and repair. Proc. Natl Acad. Sci. USA 109, E926–E933. (doi:10.1073/pnas.1106300109) 22451927 10.1073/pnas.1106300109 PMC3341051

[71] Tuynder M, Susini L, Prieur S, Besse S, Fiucci G, Amson R, Telerman A 2002 Biological models and genes of tumor reversion: cellular reprogramming through tpt1/TCTP and SIAH-1. Proc. Natl Acad. Sci. USA 99, 14 976–14 981. (doi:10.1073/pnas.222470799) 10.1073/pnas.222470799 PMC137530 12399545

[72] Telerman A, Amson R 2009 The molecular programme of tumour reversion: the steps beyond malignant transformation. Nat. Rev. Cancer 9, 206–216. (doi:10.1038/nrc2589) 19180095 10.1038/nrc2589

