# Supplementary material for: An in vivo genetic screen in Drosophila identifies the orthologue of human cancer/testis gene SPO11 among a network of targets to inhibit lethal(3)malignant brain tumour growth
Source: Open Biol. 2017 Aug 30;7(8):170156. doi: 10.1098/rsob.170156 (PMC5577452; doi:10.1098/rsob.170156)
Supplement: Supplemental Figures and figure legends 1 to 6 [file rsob170156supp1.pdf]

An *in vivo* genetic screen in *Drosophila* identifies the ortholog of human Cancer/Testis gene *SPO11* among a network of targets to inhibit *lethal(3)malignant brain tumour* growth.

Fabrizio Rossi[1,3], Cristina Molnar[1,3], Kazuya Hashiyama[1,3], Jan P. Heinen[1,3], Judit Pampalona[1], Salud Llamazares[1], José Reina[1], Tomomi Hashiyama[1], Madhulika Rai[1], Giulia Pollarolo[1], Ismael Fernandez[1] and Cayetano Gonzalez[1,2,4]

Keywords: l(3)mbt, Drosophila, malignant growth, cancer testis antigens

[1] Cell Division Group, Institute for Research in Biomedicine (IRB Barcelona), The Barcelona Institute of Science and Technology, Baldiri Reixac, 10, 08028 Barcelona, Spain.

[2] Institució Catalana de Recerca i Estudis Avançats (ICREA), Passeig Lluís Companys, 08010 Barcelona, Spain.

[3] Equal contributors.

[4] Corresponding author, <gonzalez@irbbarcelona.org>

doi: 10.1098/rsob.20170156

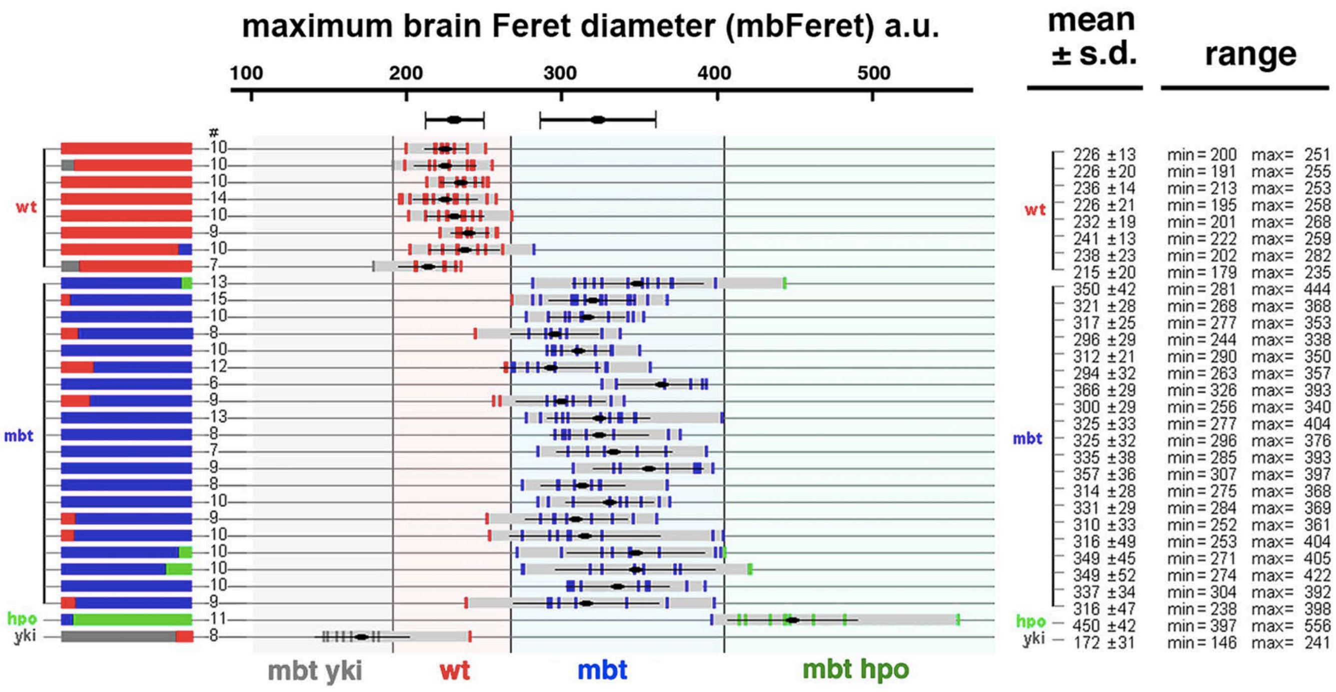

Figure S1. Size distribution of wild-type and mbt larval brains. Scattered plots showing maximum brain Feret diameters (mbFeret) of individual larval brains together with the corresponding mean, SD, and range for a series of wild-type, mbt, mbt yki, and mbt hpo samples. Each color-coded vertical line represents a single brain. Grey, red, blue, and green correspond to brains that are smaller than wild type, wild-type like, mbt-tumor like, and larger than mbt, respectively. Mean and SD for the pooled wild-type and mbt populations are shown at the top of the plot. wt= w^{1118}.

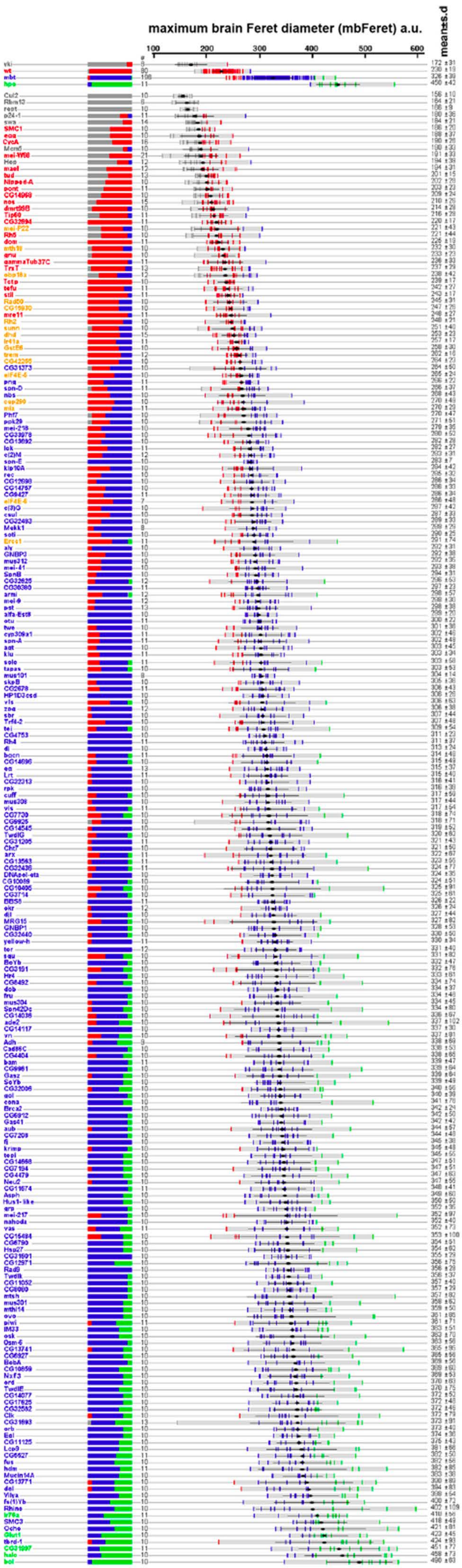
maximum brain Feret diameter (mbFeret) a.u.
100
200
300
400
500
600
mean±s.d
#

Figure S2. Plot of mbFerets obtained in the candidate screen. Color code indicates brain size class: smaller than wt (grey), wt size (red), mbt size (blue) and larger than mbt (green). A condensed plot of mbFerets together with the number of brains plotted for each experiment are shown at the left. Actual mean and SD values are shown on the right.

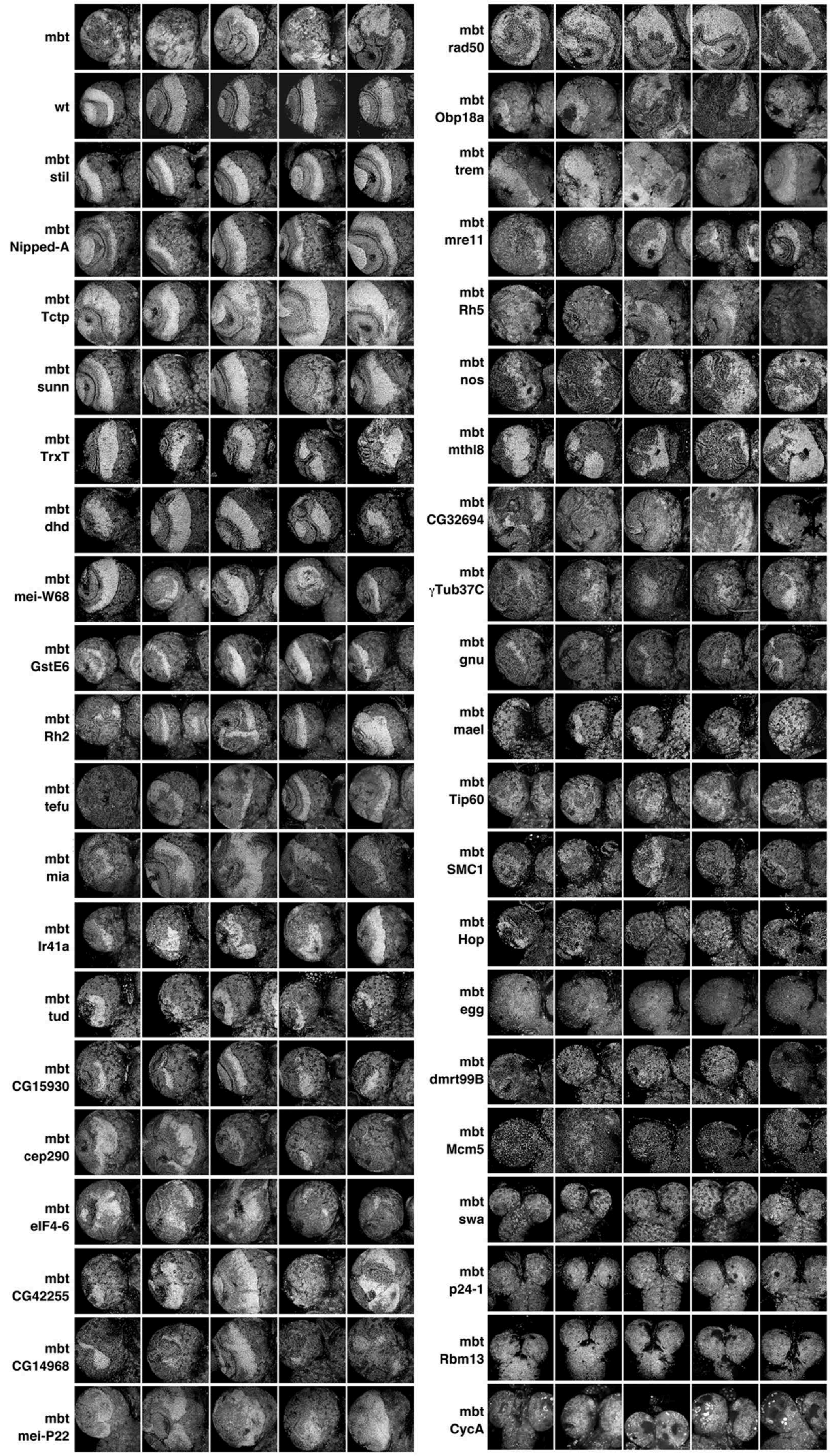

Figure S3. The phenotypic effect of mbt suppression. Array of confocal ventral sections of five DAPI stained optic lobes from each of the mbt-SPRs identified in the candidate screen. wt= w^{1118}. Scale bar=50 μm.

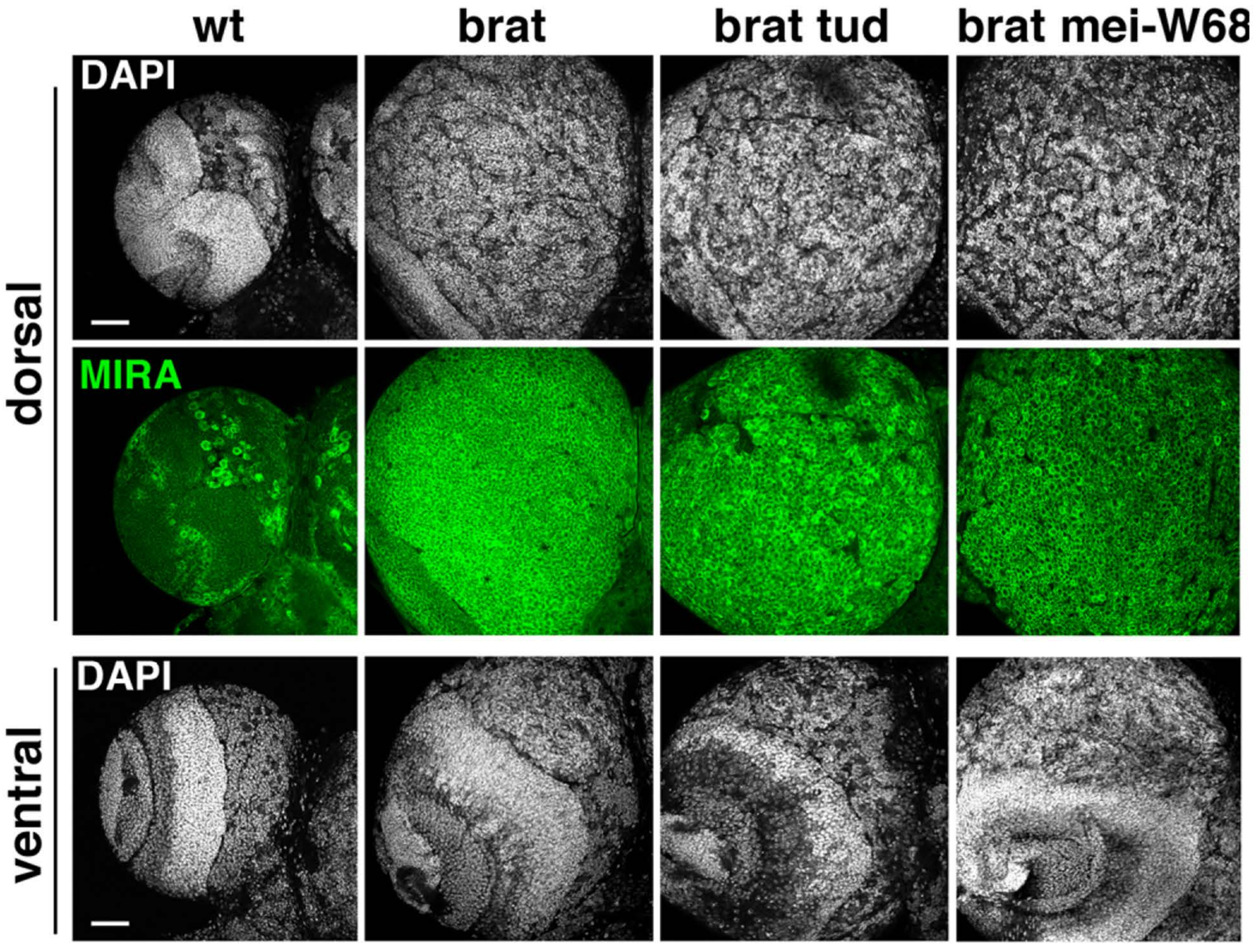

Figure S4. The mbt-SPRs *tud* and *mei-W68* do not suppress brat tumor growth. Confocal images of optic lobes from *wild type*, *brat*, and *brat* depleted for either *tud* or *mei-W68*. Dorsal sections stained with DAPI and anti-Miranda (MIRA) antibody reveal that expansion of the brat tumor mass is not curtailed by depletion either *tud* or *mei-W68*. Ventral sections show that the neuroepithelium and adjacent areas on the ventral side that are not yet reached by tumour mass. wt= w^{1118}; brat = *w; Ubi-Gal4, UAS-Dcr2/+; UAS-brat*^{RNAi}/+; brat tud = *w; Ubi-Gal4, UAS-Dcr2/+; UAS-brat*^{RNAi}/*UAS-tud*^{RNAi}, brat mei-W68 = w; *brat*^{k06028} *meiW-68*^{1} / *brat*^{k06028} *Df(2R)BSC782*. wt= w^{1118}*.* Scale bar=50 μm.

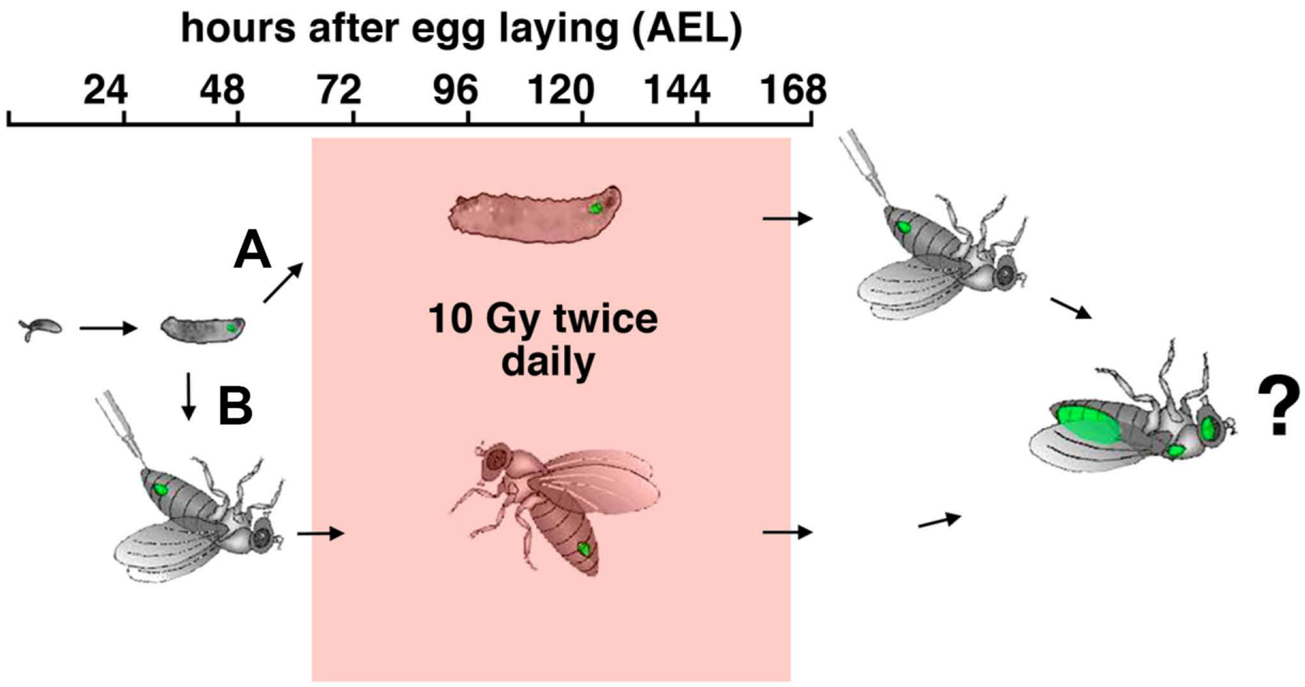

Figure S5. X-ray treatment strategies. Larvae were allowed to develop under standard laboratory conditions until 66-90h AEL. Then a twice-daily dose of 10 Gy was delivered until 143-167h AEL either (A) to the entire larvae or (B) to an adult host implanted with the larval optic lobes. Brain lobes from irradiated larvae (A) were implanted to adult hosts at the end of the X-ray treatment (168 EAL). Shaded brown background indicates the window of X-ray treatment.

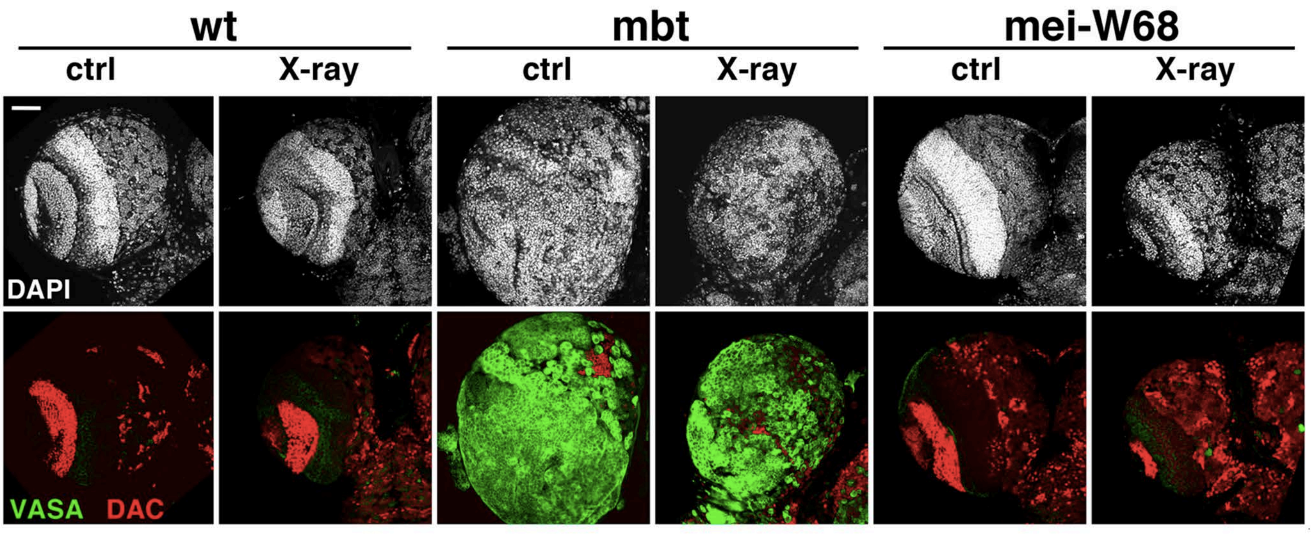

Figure S6. The effect of X-rays in *wild type*, *mbt, and mei-W68* larval brain anatomy. Confocal ventral sections of control (ctrl) and irradiated (X-ray) *wild type, l(3)mbt*^{ts1}, and *mei-W68*^{1} larval optic lobes stained with DAPI (white), anti-DAC (red) and anti-VASA (green) antibodies. wt= *w*^{1118}. Scale bar=50 µm.
